# Dr. Gairdner on Iodine

**Published:** 1824-06-01

**Authors:** 


*Essay on the Effects of Iodine on the Human Constitution; with Practical Observations on its Use in the Cure of Bronchocele, Scrophula, and the Tuberculous Diseases of the Chest and Abdomen.* By W. GAIRDNER, M.D. Octavo, pp. 64. London 1824.

SINCE we noticed the subject of Iodine, in our Number for March 1823, (Vol. III. p. 757) in the most comprehensive memoir that had, at that time, been laid before the profession in this country, with the exception of a few meagre cases in the public journals and transactions, and the unsatisfactory trials detailed in Dr. Baron's book, (see same Vol. p. 785) little additional information has been given to the English public respecting this powerful medicine. Since that time, we have ourselves used it—and seen it used—much; and we have had access to the records of a continuous and extensive practice with it, more especially in cases of bronchocele, by a physician resident in the western parts of Sussex, where this disease is particularly prevalent.

With these various stores of knowledge in our possession, we must confess that the pamphlet of Dr. Gairdner has appeared to us somewhat meagre and unsatisfactory, possessing not much original matter that could entitle it to claim the notice of the profession, and presenting what it does possess, in a manner that is neither very perspicuous nor interesting. Indeed, to such as have read our article above alluded to, we are not sure that the Essay of Dr. G. will convey much new information; but, as it certainly contains a general account of the mode of administering this remedy, and of the cautions necessary during its administration, in an accessible, if not a very lucid form,—and as we have reason to believe that the employment of this medicine is much on the increase,—we feel it our duty to recommend the work to all those who are disposed to try the remedy, and who are not already in possession of any other guide. It is especially as exposing the dangers to be apprehended from the incautious use of iodine, that we consider this pamphlet as at all likely to be useful,—and it is under this point of view particularly, that we would recommend it to the notice of the reader. "Certain statements (says Dr. G.) have gone forth to the world of the great benefits to be derived from the use of iodine, while the history of its dangers has been most unaccountably withheld. It is in order to fill up this hiatus, and at the same time to direct particularly the attention of practitioners to the proper manner of using it, with a view to its good effects, that this Essay is witten."—*Introd. p.* vii. To page 763 of our third volume, we beg to refer for a concise statement (from Brera) of the poisonous effects of iodine when incautiously administered; and we are sorry to be obliged to add that, with the single exception of any fatal event, we have repeatedly witnessed nearly all the effects there mentioned, in our own practice. In fact, we are anxious here to state decidedly and explicitly, as the result of personal observation, that iodine is a most powerful modifier of the phenomena of animal life, but, at the same time, a most dangerous, and (were it not for the hopes we entertain, that some safer rules for its administration may yet be discovered) we would add, most untractable medicine. Our employment of it has principally been in cases of bronchocele, in the speedy dissipation of which, whether taken internally or used externally, its powers are most strikingly conspicuous; although we must add, that, in the doses we have found it safe to give, the dispersion of the tumours has generally required much longer time than is stated in the Memoirs of Coindet, and in the work before us. In many cases, we have certainly found a very remarkable diminution of size within the first fortnight; and in some very slight examples, the swelling has totally disappeared within the first two months: but, generally speaking, in large and old cases, several months have commonly elapsed before any very material impression has been made; and, in more than one instance, twelve months have passed away, under the almost constant use of the remedy, before the disease has been completely eradicated. In several instances, indeed, after proceeding favourably for some time, all further progress has been interrupted notwithstanding the continual application of the former means; while, in others, the speedy supervention of bad symptoms has caused the treatment to be repeatedly stopped, and finally abandoned, when only small progress had been made towards the dispersion of the tumour. Of the two modes of using the iodine, viz. the internal and external, we are disposed, on the whole, decidedly to prefer the latter, more especially in the cure of bronchocele; although we can assert, from considerable experience, that it produces its peculiar deleterious effects on the system quite as certainly, though, perhaps, not so readily, in the one way as the other. These effects are sufficiently well described in the pamphlet before us, as, also, in the works of Coindet and Brera formerly reviewed. Those more commonly observed by us have been—head-ach—pains in the limbs and stomach, with ravenous appetite—palpitation of the heart, with great restlessness and nervous flurry—general tremors—general debility—permanently quick, strong, and hard pulse, with great emaciation, &c. In one or two cases we have had reason to suspect the supervention of organic disease of the heart (hypertrophia) as a consequence either of the direct stimulus imparted by the medicine to this organ, or of a sort of transference of nutrition to it, from the decreasing thyroid. All these effects we have witnessed from doses of the medicine much smaller than those recommended by the present author, and most others who have written on the subject as well in this country as on the continent; and we have no hesitation in asserting, that a considerable experience of the effects of it, *when genuine*, will convince any one that the doses above alluded to are much too large for safety. We have said *when genuine*, because we believe that this, like most other concentrated chemicals, is rarely to be had in a pure or trust-worthy state from common chemists. The medicine used by us has been uniformly manufactured by Mr. Battley; and we can confidently recommend the various preparations of it by that scientific chemist as entirely to be depended on, at least for purity and strength. In our first trials, we used much larger doses than we now deem safe; and although, in some few cases, we found the system but little impressed by it, we soon felt it necessary to administer it with a much more cautious hand. The preparations commonly used by us have been, for internal use, the tincture, the solution of the hydriodate, and the ioduretted solution; and externally, an ointment made either with the simple iodine, or with the hydriodate. (See Med.-Chir. Rev. Vol. III. p. 761.) Of the liquid preparations, we now consider it unsafe to give more than from five to eight drops three times a day, for a maximum dose; and we limit the use of the ointment to one application daily, or every other day. It is no doubt, true, that doses three, four, or five times as large as these may be given, in most cases, with apparent safety for some time;—and in some cases, we know, with actual safety, for a long time;—but we decidedly re-assert it, as the result of our experience, that these large doses of *the genuine drug* cannot be given generally without the greatest danger; and we conjure the younger and more zealous portion of our readers, rather to forego entirely the use of the remedy than to administer it in such quantities as we know they will ultimately repent of.

In arguing from the old medical adage "nil prodest quod non potest lædere idem," we ought to expect much benefit from the use of iodine; and we confess that we are very unwilling to forego the hope of this benefit being eventually secured to medicine, by the further prosecution of the subject of its mode of acting on the living system. It is to this point, more particularly, that we would beg leave to direct the attention of practitioners; as, until more precise rules for its administration are established, we are convinced that its general adoption into practice will be infinitely more injurious than beneficial to humanity. We dwell the more on this, as we have reason to believe that we ourselves, by our former reviews, have been the means of leading many to adopt the use of iodine; and because we fear that the direction given to its employment in Dr. Baron's late work, is likely to tend to an extension of its use in a manner that must prove injurious in the highest degree. So far, indeed, from iodine being generally useful in cases of phthisis pulmonalis—at least, in the large doses recommended—we are convinced that it must tend directly to precipitate the progress of that almost or altogether incurable malady; and we here loudly enter our protest against its employment, in this and other internal diseases, until such times as its virulence has been tamed by an improved mode of administration, and its powerful influence on the system rendered subservient to the removal of disease *only*. It is indeed true, that it is only by trials on the human subject, that the real value of this or any other medicine can be ascertained; and it must be admitted, that it is in such hopeless diseases as consumption that such trials may with most propriety be made; at the same time, as no man could be justified in hazarding unnecessarily the present degree of vigour of his patient, we would recommend all experiments with iodine in internal diseases, and especially in phthisis, to be made with extreme caution, and with very minute doses of the medicine.

The following extracts from Dr. Gairdner's pamphlet will illustrate this subject still further.

"Some time after the introduction of iodine into practice, a few cases of severe spasmodic affection of the stomach and bowels occurred. They were attended with violent and incessant vomiting, excruciating pain of stomach and bowels, strong spasms of the back and legs. The tongue was commonly furred, and the bowels sometimes violently purged, at other times obstinately constipated. The pulse was generally extremely frequent, small and depressed—the eyes sunk and hollow—the countenance ghastly and pale. These accidents were usually imputed by the patients to the iodine they had taken. The physicians by whose advice the medicine had been given, would not allow this origin of the disease, till a repetition of similar cases determined that the sufferers were right.

The vomiting, pain of the bowels, and the cramps of the legs, are extremely severe. They are also with the greatest difficulty allayed, continuing sometimes for many days, and renewed during weeks, and even months, after taking food. The legs sometimes swell in the first instance, and afterwards become rapidly thin and meagre. There is another symptom, which, though common to almost all diseases, is peculiarly the sign of this. The emaciation which attends this irregular action of iodine is so rapid and so extreme as to strike terror into the minds both of patients and physician." 7.

"There is an effect of iodine which is so extremely common, when the remedy has been pushed to an overdose, that it deserves to be noticed at greater length. The anxiety and depression of spirits are so great and persevering as to warrant my considering them as the peculiar effect of iodine, and not the consequence of the great debility which attends the violent and inordinate action of this medicine on the constitution. It is an affection very different from hypochondriacal melancholy, inasmuch as it dwells principally on the present and has no reference to the future. Patients have generally described it to me as a sense of sinking and faintness, which were peculiarly oppressive, and I have heard them complain of it while suffering the most intense pain, as the part of the complaint which was yet the most difficult to bear. This symptom is an almost constant attendant on the violent action of iodine on the system, and frequently makes its appearance in a lesser degree when the medicine acts in a kind and salutary manner." 13.

"The nervous and muscular systems are peculiarly exposed to the irregular action of this medicine. In certain persons, indeed, of peculiar habits of body, it cannot be exhibited so as to affect the constitution in any manner, without in some shape or other producing unpleasant nervous symptoms, such as dimness of vision, indistinct hearing, fallacious touch, insomnia, breathlessness, palpitation, and all the countless forms of inward nervous derangement. But the symptom to which we shall more particularly confine our attention, is a degree of tremor which generally comes on when the patient is under the full constitutional influence of iodine. This symptom may be reckoned a good gauge of the degree of nervous excitement which has taken place, and it is seldom or never absent when that excitement has proceeded to any considerable degree. It generally begins by a slight trembling of the hands, resembling that which takes place from the poison of lead; and if the medicine be incautiously continued, the larger muscles of the arms, legs, and back become affected. When in this state, the patient can with difficulty walk, and his progression is a tottering uncertain motion. He cannot carry any thing straight to his mouth, but the hand moves in a zig-zag manner, and with difficulty arrives at the mouth at last. This complaint is generally attended with a hurried circulation, and a small thready pulse. There is commonly great suffering at stomach and confined bowels. When nervous affection first appears the medicine must be most diligently watched, and if the symptoms seem to increase, its use should be instantly put a stop to. If rashly persevered in, the symptoms I have described above will certainly be excited, and then it is vain to withdraw the medicine; the complaint goes on progressive for weeks and months, even though its exciting cause be abstracted; and when it does at last begin to diminish, the amendment is so slow and gradual that the patient is scarcely conscious of the relief he receives. I saw two cases of this kind with Dr. Peschier of Geneva, in which the patients had suffered more than twelve months, and yet their sufferings had undergone little mitigation. It is of some importance not to provoke a complaint with so much difficulty allayed; and no one who has not seen it can have an idea of the slow and imperceptible degrees by which it steals on the patient. Its first advances generally escape his observation as well as that of his physician. A slight trembling of the fingers, quivering of the eyelids, occasional subsultus of the tendons of the fingers, arms, and legs, are generally the first symptoms observed, and it behoves us to be constantly on the watch for them. I have always obliged my patients to raise an empty glass or any light object to the head. By this means the smallest degree of unsteadiness in the hand will commonly be detected. I recommend a light object to be used for this purpose, because a heavy one tends to give steadiness to the muscles and to disguise the complaint." 16.

"The affection of the alimentary canal is plainly to be ascribed to the acrid operation of iodine on its mucous membrane. I have never witnessed it in any considerable degree when this medicine had not been taken internally. But I have seen slight pains of stomach, accompanied with copious bilious evacuations, attend its external use. These never proceed to the degree of violence which marks the internal exhibition. Indeed, it is rare to see them in any considerable degree disturb the comfort of the patient. It is not thus when taken into the stomach. I have never seen any disease of the bowels which more closely resembled the terrific descriptions given by the physicians of India, of the sufferings from the cholera of that country. Yet no medicine varies more in its effects than this. Some persons take it in large doses for a great length of time with perfect impunity; while others, from that peculiar, undescribed and unintelligible state of constitution, called by physicians an idiosyncrasy, are speedily and violently affected by very small doses" 20.

"It is a much more difficult task to discover a probable explanation of the manner in which iodine disturbs the actions of the nervous system." 21.

Perhaps the undoubted, and indeed very obvious affection of the mucous membrane of the stomach and bowels, which exists both in the slightest temporary disorder, and in the most prolonged chronic cases, may be sufficient to account for most, if not all, the other symptoms, by means of the chain of pathological sympathy, which Broussais, more especially, has shown to extend from this centrical focus, embracing and involving every point, fibre and function of the system.

Dr. Gairdner affords us little or no assistance in the cure of this most distressing factitious disease; and, indeed, this will not surprise them who consider the untractable nature of most of those affections which appear to have their site in the same organic texture, and to consist essentially in some analogous pathological modification of the living fibre. Under this head we would class numerous cases of *fever*, typhus and others—choleras—certain diarrhœas, dysenteries, and lienteries—a large class of affections usually ranked under the head of dyspepsia, biliary derangements, &c.—diabetes—certain cutaneous diseases, (so called,) &c. &c. &c. all of which, we have little hesitation in saying, are rather injured than benefited by the prevalent modes of practice in England; and all of which, even under the most rational treatment, are found to be obstinate and unmanageable in the highest degree.

Our author, in these cases, recommends, in the first stage, opium, hemlock, or hyoscyamus, with diluting and emollient injections, and more especially the warm bath; these may give temporary relief. In the second or chronic stage, when ulceration of the bowels has occurred, we believe that it is only by the strictest regimen, long continued, and aided occasionally by the mildest laxatives, that any essential benefit is to be looked for. In these particulars, we are pleased to find our own experience coincide with that of Dr. G's; with the exception, however, of *bitters*, which we have found hurtful in every variety of the complaint.

"I have tried various bitter and astringent medicines in union with opium, but have found them uniformly injurious during the first stage of excitement and exacerbation. Afterwards, when the disease has in some degree abated, this class of medicine will be found useful. I cannot too strongly caution my readers against the use of purgatives in such cases. However gentle they may be, their effect is uniformly and most decidedly noxious. In the first and acute period of this affection of the alimentary canal, it is almost impossible to quiet the disturbance which a purgative occasions. A remedy which ought never to be neglected is the warm bath. It will be found a most powerful coadjutor in restraining the violence of the spasms, and in moderating the perturbed action of the stomach." 25.

Dr. Gairdner adopts the common opinion of iodine being a direct stimulant of the absorbent system—an opinion which seems almost entirely founded on its striking power in producing emaciation. This conclusion is clearly illogical, unless it can be shown that it does *not* produce any other effects on other textures or functions, by which the phenomena may be explained. One interesting fact, certainly favourable to his opinion, and which is new to us, is mentioned by the author—viz. the action of the medicine on the system being quickened by blood-letting. "Whenever the medicine is slow in its operation, provided the vessels be full and plethoric, I desire a little blood to be taken away from the arm, and I almost invariably find the action of the medicine much quickened. I have sometimes, also, thought that the cases, in which blood was taken away, were cured more easily and with less suffering than others." 38.

For the mode of administering iodine we must refer to Dr. Gairdner's book, or to our own Review, already so often alluded to—cautioning our readers against the use of the large doses therein recommended. Dr. G's ointment is formed by rubbing up half a dram of the hydriodate of potass, with an ounce and half of lard; and of this half a dram is rubbed on the bronchocele night and morning. For internal use he also prefers the salts to the pure iodine. He dissolves half a dram of the hydriodate in an ounce of distilled water, and gives of this at first ten drops, "augmented gradually to twenty, and, very seldom, to twenty-five."

Dr. Gairdner's little work concludes with some observations on the use of iodine in scrofula, phthisis, and mesenteric decline, in all of which he thinks very favourably of it. For his opinions and remarks we must refer to the original. We cannot, however, help being struck with the unsatisfactory evidence on which both he and Dr. Baron seem to consider its efficacy as, in some degree, confirmed by experience, in the removal of internal diseases.

In conclusion, we would once more earnestly call the attention of our readers to the cautious study and use of iodine, with a view to the discovery of the particular action on the body, on which its beneficial effects depend; and with the view to the establishment of more certain and safer rules for its administration than we yet possess. At present, it is like arrows in the hand of a child, or like fire in the hands of a fool, more likely to do harm than good. Still we are most unwilling to forego the expectation that its great powers may yet be rendered entirely subservient to useful purposes, as the arrows or the fire, when wisely directed. Whether this beneficial improvement is to be looked for to the modifying influence of other medicinal agents, to the diminution of its dose, or to any other cause, we do not pretend to divine: whenever or however it is attained, we shall be satisfied. Our phrenological friends will be aware that even a large original dose of the propensities of *combativeness* and *destructiveness*, is rather desirable than otherwise, as adding much to the usefulness and detracting nothing from the amiableness of a character—provided they are duly counterbalanced by a proportionate share of the milder sentiments: in like manner, we confess, that it is the very decided activity of iodine as a modifier of the phenomena of life, that makes us anxious to retain it among the class of medicinal agents, and leads us to hope that means may yet be found to turn all its evil to good—to strip it of its virulence, without diminishing its vigour.

---

VIII.

